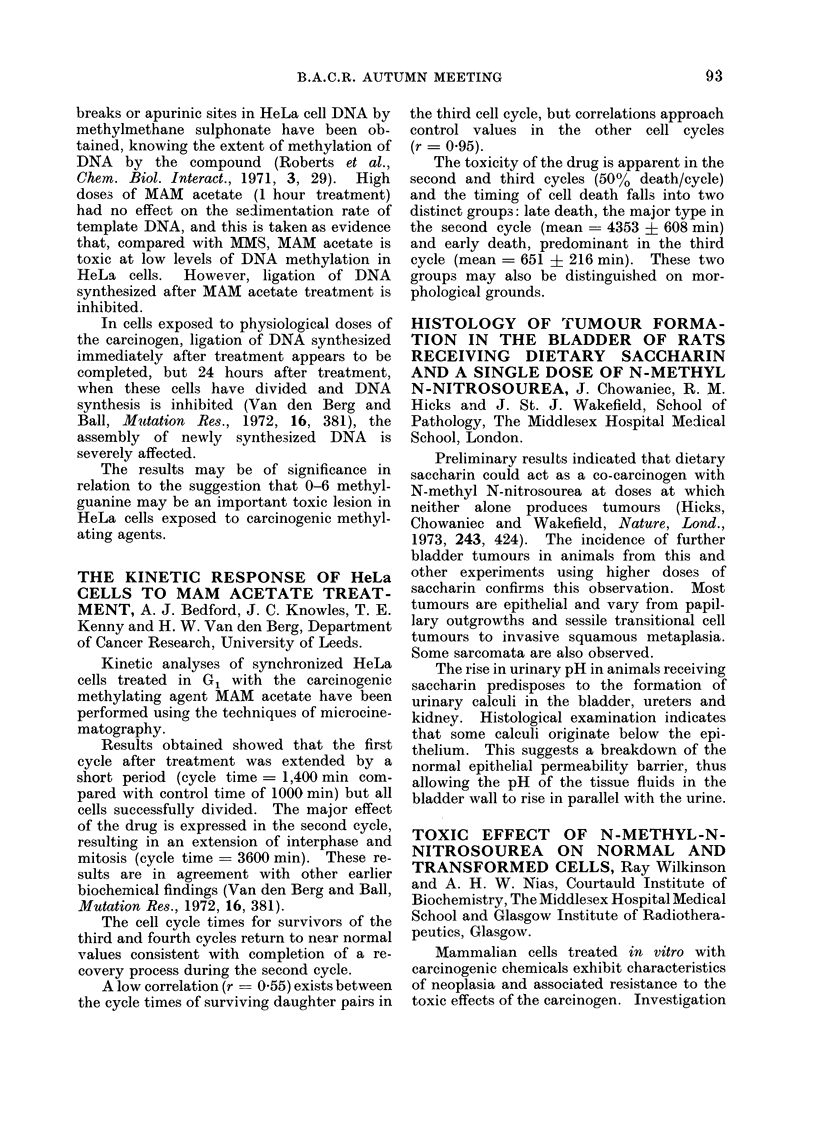# Proceedings: Histology of tumour formation in the bladder of rats receiving dietary saccharin and a single dose of N-methyl N-nitrosourea.

**DOI:** 10.1038/bjc.1974.18

**Published:** 1974-01

**Authors:** A. J. Chowaniec, R. M. Hicks, J. S. Wakefield


**HISTOLOGY OF TUMOUR FORMATION IN THE BLADDER OF RATS RECEIVING DIETARY SACCHARIN AND A SINGLE DOSE OF N-METHYL N-NITROSOUREA,** J. Chowaniec, R. M. Hicks and J. St. J. Wakefield, School of Pathology, The Middlesex Hospital Medical School, London.

Preliminary results indicated that dietary saccharin could act as a co-carcinogen with N-methyl N-nitrosourea at doses at which neither alone produces tumours (Hicks, Chowaniec and Wakefield, *Nature*, *Lond.*, 1973, **243**, 424). The incidence of further bladder tumours in animals from this and other experiments using higher doses of saccharin confirms this observation. Most tumours are epithelial and vary from papillary outgrowths and sessile transitional cell tumours to invasive squamous metaplasia. Some sarcomata are also observed.

The rise in urinary pH in animals receiving saccharin predisposes to the formation of urinary calculi in the bladder, ureters and kidney. Histological examination indicates that some calculi originate below the epithelium. This suggests a breakdown of the normal epithelial permeability barrier, thus allowing the pH of the tissue fluids in the bladder wall to rise in parallel with the urine.